# 1099. Evaluation of the Safety and Pharmacokinetics (PK) following Administration of Single and Multiple Doses of Anti-Staphylococcal Lysin, LSVT-1701, in Healthy Adult Subjects

**DOI:** 10.1093/ofid/ofab466.1293

**Published:** 2021-12-04

**Authors:** Mary Beth Wire, Soo youn Jun, In-Jin Jany, Jun Gi Hwang, David Huang

**Affiliations:** 1 Lysovant, New York, New York; 2 iNtRON Biotechnology, Seoul, Seoul-t’ukpyolsi, Republic of Korea; 3 iNtRON, Seoul, Seoul-t’ukpyolsi, Republic of Korea

## Abstract

**Background:**

LSVT-1701 is an anti-staphylococcal phage lysin being developed for treatment of MRSA infections in combination with SoC antibiotics. The safety and PK of single ascending doses of LSVT-1701 0.1 to 10 mg/kg in healthy adult volunteers were previously described (Jun, et.al, AAC 2017;61:e02629-16). We further evaluated the safety and PK of multiple ascending doses of LSVT-1701 in healthy adult subjects.

**Methods:**

Study ITB-101-1b was a Phase 1, randomized, double-blind, placebo-controlled, multiple ascending dose study. 8 subjects were randomized 3:1 to active:placebo in each cohort. LSVT-1701 was administered as a 6 mg/kg single dose and twice daily (BID) doses of 1.5, 3.0, and 4.5 mg/kg for 4 days (24h between Doses 1-2, 12h between Doses 2-6). Study drugs were administered as a 1-hour IV infusion. Serial serum samples were collected over 24 hours following the first and last doses for measurement of LSVT-1701 concentrations by a validated ELISA method. PK analysis of LSVT-1701 concentration-time data was done using noncompartmental methods. Safety was assessed by AEs, clinical labs, vital signs, and ECG.

**Results:**

30/32 (94%) subjects completed the study. No subjects withdrew due to AEs, and there were no severe AEs, no serious AEs, and no deaths. AEs were of mild (97%) to moderate (3%) intensity and were reported by all subjects in the LSVT-1701 6 mg/kg single dose group and 1-3 (17-50%) of subjects receiving 1.5 to 4.5 mg/kg BID or placebo. The most common AEs of headache, chills, rigors, and fever generally lasted for ≤2 days with or without acetaminophen treatment, and no clinically significant changes in blood pressure, heart rate, ECG, or clinical labs (other than transient increases in CRP) were observed. Infusion site reactions (erythema, pain, induration, warmth) were observed with BID administration of LSVT-1701, but not with the single 6 mg/kg dose or placebo. LSVT-1701 exposure increased greater than in proportion to dose and t_1/2_ was concentration-dependent, increasing with higher doses. No accumulation in LSVT-1701 exposure was observed.

Summary of LSVT-1701 PK Parameters

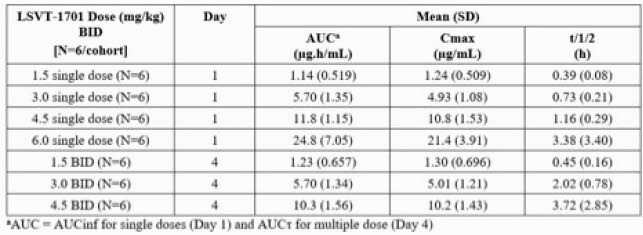

Summary of LSVT-1701 PK Parameters

**Conclusion:**

The safety and PK profile of LSVT-1701 is favorable for evaluation in patients with

*S. aureus* infections, including bacteremia and infective endocarditis, for which new treatments are needed.

**Disclosures:**

**Mary Beth Wire, Pharm#**, **Lysovant** (Consultant) **Soo youn Jun, PhD**, **iNtRON Biotechnology** (Consultant) **In-Jin Jany, PhD**, **iNtRON** (Consultant) **Jun Gi Hwang, PhD**, **Lysovant** (Consultant) **David Huang, MD, PhD**, **Lysovant** (Consultant)

